# Use of WhatsApp messaging technology to strengthen obstetric referrals in the Greater Accra Region, Ghana: Findings from a feasibility study

**DOI:** 10.1371/journal.pone.0266932

**Published:** 2022-04-28

**Authors:** Medge D. Owen, Hebah M. Ismail, David Goodman, Mariam Batakji, Sung Min Kim, Adeyemi Olufolabi, Emmanuel K. Srofenyoh

**Affiliations:** 1 Department of Anesthesiology, Wake Forest School of Medicine, Winston-Salem, NC, United States of America; 2 Winnie Palmer Hospital for Women and Babies, Orlando, FL, United States of America; 3 Department of Anesthesiology, Medical College of Wisconsin, Milwaukee, WI, United States of America; 4 Department of Anesthesiology, Vanderbilt University Medical Center, Nashville, TN, United States of America; 5 Department of Anesthesiology, Duke University Medical Center, Durham, NC, United States of America; 6 Greater Accra Regional Hospital, Ghana Health Service, Accra, Ghana; Aga Khan University, PAKISTAN

## Abstract

In Ghana, the high-risk obstetric referral system is inadequate. Delay is common and patients often arrive to receiving hospitals in compromised states. An effective referral system should include an adequately resourced referral hospital, communication across sectors, accountability, transport, monitoring capability and policy support, which are currently lacking. A pilot program was undertaken to facilitate communication between hospital staffs. Additionally, data was collected to better understand and characterize obstetric referrals in Accra. Thirteen institutions were selected based on referral volume to implement the use of pre-referral treatment guidelines and WhatsApp as a mobile technology communication platform (Platform). Participants included healthcare workers from 8 health centers, 4 district hospitals, the Greater Accra Regional Hospital (GARH), administrators, doctors from other tertiary hospitals in Accra and medical consultants abroad. Facilities were provided smartphones and guidelines on using WhatsApp for advice on patient care or referral. Data were collected on WhatsApp communications among participants (March-August 2017). During this period, 618 cases were posted on the Platform and users increased from 69 to 81. The median response time was 17 min, a receiving hospital was identified 511 (82.7%) times and pre-referral treatment was initiated in 341 (55.2%). Subsequently, data collected on 597 referrals to GARH (September-November 2017) included 319 (53.4%) from Platform and 278 (46.6%) from non-Platform hospitals. Of these, 515 (86.3%) were urgent referrals; the median (interquartile range) referral to arrival time was 293 (111–1887) minutes without variation by facility grouping. Taxis were utilized for transportation in 80.2%; however, referral time shortened when patients arrived by ambulance and with a midwife. Only 23.5% of urgent referrals arrived within two hours. This project demonstrates that WhatsApp can be used as a communication tool for high-risk obstetric referrals and highlights the need to continue to improve urban referral processes due to identified delays which may contribute to poor outcomes.

## Introduction

Ghana has made significant progress towards promoting access to obstetric services as reflected by a rapid increase in the institutional delivery rate over the past decade. Currently, 79% of expectant mothers deliver in health facilities assisted by skilled birth attendants, an increase from 54% 10 years ago [1]. Despite this improvement, significant gaps remain in the provision of quality care for high-risk obstetric patients, particularly along the referral pathway [2–6]. At least 15% of pregnancies develop complications that require advanced care, including surgery, in hospitals capable of performing comprehensive emergency obstetric care (CEmOC) [7]. The World Health Organization currently recommends that emergency obstetric services should be available within two hours of seeking care [8]. Similarly, the Lancet Commission on Global Surgery recently adopted six quality of care indicators to assess surgical readiness, among which is access to care within two hours to facilities capable of performing emergency surgery [9].

Delays in reaching appropriate health facilities contribute to poor maternal and newborn outcomes [10–13]. Inefficient referral processes can result in death during transport or soon after arrival at secondary and tertiary institutions [2, 3, 13]. As such, referral and teaching hospitals have maternal mortality rates that far exceed the national average [3, 14, 15]. In Ghana, the country action plan for the Millennium Development Goal (MGD) Acceleration Framework identified weak referral systems, particularly for managing obstetric emergencies, as one of the leading challenges to achieving the MDG for maternal mortality [16].

Numerous measures to strengthen Ghana’s referral system have been undertaken. In 2012, the Ministry of Health deployed a national policy to address delays in accessing emergency care for referred patients [17]. Other measures have included a National Health Insurance Scheme with free access to maternity care, a national ambulance service, implementation of maternal death audits and the use of audit findings to inform referral institutions, the establishment of referral ledgers, and policies to ensure that health staff accompany referred emergencies [2–4, 17, 18]. In addition, the Greater Accra Region has established a “call centre” to facilitate linkages between the source and receiving hospitals. Despite these interventions, the referral system remains challenged [2]. There are recent reports in the lay press on the “no bed syndrome” whereby referred emergencies are denied admission and care at one institution and are repeatedly referred elsewhere [2].

A way to improve the referral process is to leverage messaging technology to promote collaboration between hospitals. Smartphones, tablets, and messaging services have become commonplace. WhatsApp is a popular messaging service with over 2 billion users in 180 countries [19]. It is a free smartphone application that utilizes the internet to share text, photographs, video, documents, and voice calls. With end-to-end encryption, WhatsApp is an emerging medium for healthcare providers, especially in low resource settings, where access to landlines, computers, and other communication tools is limited [20, 21].

A feasibility project was undertaken in Ghana to utilize WhatsApp as a mechanism through which the high-risk obstetric referral process could be improved. Conceptualized by local physicians frustrated by the ineffective referral process, the idea was to create an inter-institutional communication platform that would link healthcare providers at primary level health centers and district hospitals to senior clinicians at a large regional hospital in the Greater Accra Region. The goal was to improve communication among clinicians to help identify cases appropriate for referral, guide treatment interventions, expedite inter-institutional transfers, facilitate feedback, and improve system-level accountability by including hospital and health service administrators and the national ambulance service. The intervention was thought to be a viable, low-cost solution to referral communication difficulties since smartphones and WhatsApp are commonly used throughout Ghana. Reports on the use of WhatsApp in maternal and child health literature have been limited. WhatsApp has been used to facilitate pregnancy support groups in Kenya [22], to enhance communication among maternal-fetal medicine specialists across continents [20] and to support neonatal referrals in Cameroon [23]. However, to the authors’ knowledge, only one small study exists that evaluates the use of a mobile messaging application on addressing CEmOC referral care [24]. The aim of this study was to determine the feasibility of using WhatsApp as a communication tool among clinicians as well as to understand timeliness and other characteristics of obstetric referrals to a large regional hospital in Accra, Ghana.

## Methods

### Study setting

The Greater Accra Region of Ghana comprises 3,245 km^2^ and serves 4 million inhabitants, approximately 16% of Ghana’s population [25]. The predominately urban region represents the most educated and wealthiest segment of the population [1]. The government health system is organized within a three-tier model with care escalating from primary care and community health centers, to district hospitals, then to regional and teaching hospitals [2, 3, 17]. Additionally, there are numerous smaller private facilities with varying capabilities. The Greater Accra Regional Hospital (GARH) is a major obstetric referral facility in the capital city and one of the largest regional hospitals in Ghana. The hospital conducts approximately 8,000 deliveries per year, of which 70% are high-risk referrals [26]. The GARH receives obstetric patients from private hospitals, health centers, district hospitals, and other secondary level institutions across the metropolitan area, some of which also provide CEmOC.

### Intervention

The Kybele Referral Platform (Platform) was created as a closed WhatsApp (Meta, Mountain View, CA) group consisting of obstetricians, midwives, administrators, and other representatives from eight primary health centers, four district hospitals and GARH. In addition, Ghana Health Service directors and consultant physicians from the United States and England affiliated with the project were included. The facilities represented six districts in the Greater Accra Region and were high volume referral sources to GARH. The participating facilities included Kaneshie Polyclinic, Amanfron Health Center, Mamprobi Polyclinic, Madina Kekele Clinic, Madina Rawlings Park Clinic, Nima Government Hospital, Adabraka Polyclinic, Osu Government Maternity Home, Achimota Hospital, Ga South Municipal Hospital, Pentecost Hospital, Maamobi General Hospital, and GARH.

One smartphone (Alcatel Onetouch Pixi 4) was distributed to each participating facility obstetric unit for engagement on the Platform. Participants could also elect to join the Platform using their personal smartphones. Two project leads (EKS and AO) became Platform administrators who added the mobile numbers of invited participants, hospital administrators and other focal persons, thus creating a private user group. Facility participants attended a half day informational session on January 17, 2017. Participants were selected by their hospital managers and included doctors, midwives, and health information officers. The session included instructions and role play on how to appropriately post cases and emphasized the importance of maintaining patient privacy, timeliness in responding to a post and ground rules for respectful communication in the user group. A protocol book to aid in diagnosis and pre-referral treatment protocols was also distributed. Topics included indications for referral, high alert situations, critical logistics for emergency care, and treatment protocols for common pregnancy related complications. The Platform launched across 13 facilities on March 1, 2017.

Participants posted deidentified patient information, including gestational age, current condition, investigations conducted, treatments initiated, and the reason for potential referral. The Platform prohibited use of patient names, photographs, or other identifying information. Upon reviewing the clinical data and referral request, clinical teams at GARH, or any other facility, could advise the initial team on what investigations to perform, treatments to start, and the ability to accept transfer, if appropriate. Any group participant could post a case or respond to a posting day or night and from any location worldwide.

### Data collection and analysis

Transcripts of all WhatsApp cases posted from March through August 2017 were exported and printed. The following information was collated and manually re-entered into Microsoft Excel (Microsoft, Redmond, WA) for analysis: referring hospital, date, time of posting, time of response, time of transfer decision, diagnosis, the reason for referral, maternal and/or fetal indication, treatment rendered, destination hospital, and outcome.

In addition, data were collected on obstetric referrals to the GARH from September 1 to November 30, 2017 to further elucidate the nature of referrals from both participating and non-participating hospitals. Two hired data collectors manually extracted referral information from patient charts and logbooks within two weeks of referral. Data were entered into Microsoft Excel (Microsoft, Redmond, WA) and crosschecked for accuracy by separate members of the research team. Patient data included maternal age, gravida, parity, gestational age, educational status, labor characteristics, time of arrival, delivery mode, and maternal and fetal outcomes. Referral data included the name of the source institution, reason for referral, mode of transport, persons accompanying the patient, and the time interval from referral to arrival. Global Positioning System (GPS) coordinates were gathered for the source facilities using Google Maps software (Google, Seattle, WA) that is publicly available. Care was taken to identify each facility through a Google search on the software or by finding the location on a map with the help of an author with local expertise (EKS). The GPS information was mapped using Tableau Public Version 9.1 software (Tableau, Seattle, WA). The GPS data were overlaid on a map of Accra provided by Mapbox (www.mapbox.com) using open-source utilities that are free to use with proper attribution.

Data were transferred to Stata version 15.1 (Stata Corp, College Station, TX) for analysis. Data are presented as average ± SD, number (percent), or median (IQR). Where applicable, categorical variables were compared using the Pearson’s Chi-square test and continuous variables were analyzed using Student’s t-test. A p-value of 0.05 was considered statistically significant. When the amount of time required for referrals was analyzed, it was treated as a continuous variable and analyzed using the Wilcoxon Rank Sum test for non-parametric distributions.

The Ghana Health Service (Ref. No. GHS/DGS/K-6) and Wake Forest University Health Sciences (IRB00047565) granted ethical approval for this work. This research met institutional review board criteria for a waiver of consent according to 45 CFR 46(d).

## Results

### WhatsApp platform utility

From March through August 2017, 618 cases were posted on the Platform, representing roughly 25% of the usual number of referrals. During that time, the number of Platform users increased from 69 to 81; the additional participants included physicians from other hospitals that acted as receiving hospitals, the national ambulance service, Ghana Health Service directors and members of the GARH neonatology unit. Four hundred nine (66.1%) cases were posted during the day (08:00–19:59) and 209 (33.8%) were posted at night (20:00–07:59). Overall, the median (IQR) posting to response time was 15 min (5–40 min) and varied from 13 min (5–36 min) during the day to 18 min (6–53 min) at night. The minimum response time was 1 minute; however, occasionally, posted cases did not get a response for hours (max 16 hr 11 min). Generally, if a case was severe and there was no timely response, the posting hospital would repost the case asking for help.

Of the 618 cases, 304 (49.2%) were posted for both maternal and fetal indications, 187 (30.3%) were for maternal reasons, 77 (12.5%) were for fetal reasons, 40 (6.5%) were for newborns, and the remaining 10 (1.6%) were unrelated to a referral. Non-patient related postings alerted Platform users of equipment malfunctions, such as a faulty anesthesia machine, or the unavailability of oxygen or blood, thus temporally deferring referrals. The most frequent conditions posted included: hypertensive disorders of pregnancy (226; 36.6%), labor dystocia (151; 24.4%), premature labor (148; 23.9%), fetal compromise (86; 13.9%), and acute maternal hemorrhage (62; 10.0%). Five hundred eleven (82.7%) of the posted cases had a receiving hospital identified on the Platform prior to transfer. Of these, the GARH received 312 (61.0%), other district level Platform hospitals received 97 (19.0%), other secondary or tertiary level non-Platform hospitals received 48 (9.4%), and in 54 (10.6%) cases patients were deemed too advanced in labor for transport and subsequently managed on site.

Treatment was initiated in 341 (55.2%) cases prior to referral using project-established guidelines. Other Platform uses included the exchange of advice on treatment (91; 14.7%) and investigations (46; 7.4%), help with diagnosis (4; 0.6%), and to request (28; 4.5%) or to provide feedback (47; 7.6%) on outcomes.

### Characterizing referrals to GARH

**Fig 1:**
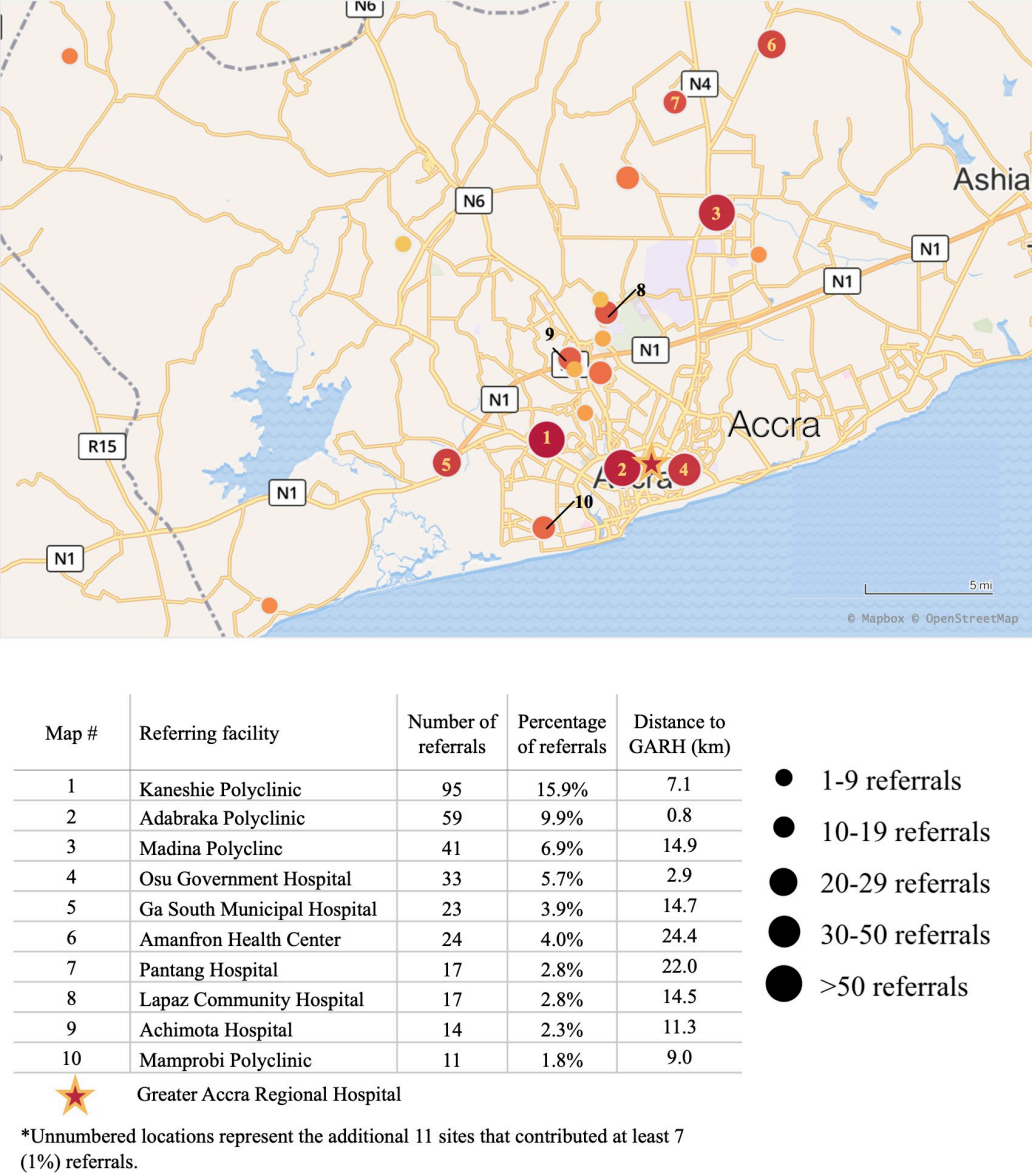
Map of Accra, Ghana, demonstrating the location and volume of obstetric referrals from the ten leading referral facilities to the Greater Accra Regional Hospital. The figure was created using Tableau data software and the map was generated using Mapbox open-source utilities. The Map image used open-source software found at https://www.mapbox.com/about/maps/ [mapbox.com], with data from http://www.openstreetmap.org/about/ [openstreetmap.org]. Everyone is welcome to improve the map here: https://www.mapbox.com/contribute/[mapbox.com].

The GARH had 1266 referrals from September 1 to November 30, 2017. Data were collected on 652 of these; however, 55 were excluded due to incomplete referral and arrival times. Data were analyzed for 597, representing 47.5% of referrals during the study timeframe; 319 (53.4%) were from Platform and 278 (46.6%) were from non-Platform institutions. There were 370 (62.0%) referrals from health centers; 85 (14.4%) from district hospitals; 16 (2.7%) from tertiary or teaching hospitals; 120 (20.3%) from private hospitals, and 6 (1.0%) were from unknown facilities. Six (1.0%) patients had two referral points. Referrals emanated from 114 institutions; Fig 1 shows the locations of the ten highest volume source hospitals, 8 of which were Platform institutions. More referrals were made and received during the day (8:00–19:59); however, a significant proportion of these arrived at night (20:00–7:59) [referral time: day 444 (74.4%), night 153 (25.6%) vs arrival time: day 371 (62.1%), night 226 (37.9%); p<0.001)]. This was consistent across Platform and non-Platform facilities.

Patient demographic information is shown in Table 1. The distance traveled, mode of transportation, and accompanying personnel are shown in Table 2. Platform hospitals were significantly closer in proximity to GARH than were non-Platform hospitals (p<0.01). The most distant referral site was 145.8 km from GARH. As shown, taxi was the predominant mode of transportation. There were no differences in mode of transport among facility types.

**Table 1:** Patient data and education of referred obstetric patients.

**Patient Data**	**Total (N = 597)**	**Platform (N = 319)**	**Non-Platform (N = 278)**
Age (yr)	29.7 ± 5.6	29.4 ± 5.7	30.0 ± 5.4
Range	15.0–45.0	16.0–43.0	15.0–45.0
Gravida	3.0 ± 1.7	3.1 ± 1.7	3.0 ± 1.7
Parity	1.5 ± 1.4	1.6 ± 1.4	1.4 ± 1.4
Gestational age (wk)	37.5 ± 7.5	37.8 ± 5.4	37.0 ± 9.3
**Education**	**Total**	**Platform**	**Non-Platform**
Primary	55 (9.3)	25 (7.9)	30 (10.9)
Junior high school	230 (38.9)	135 (42.6)	95 (34.7)
Senior high school	184 (31.1)	107 (33.8)	77 (28.1)
Tertiary	115 (19.5)	46 (14.5)	69 (25.2)
Uneducated	7 (1.2)	4 (1.3)	3 (1.1)
Not available	6	2	4

Data presented as mean ± SD or number (percent). There are no differences between Platform and non-Platform institutions.

**Table 2:** Distance, mode of transportation and accompanying person.

**Distance from GARH (km)**	**Total**	**Platform**	**Non-Platform**
<9.9	257 (43.0)	199 (62.4)	58 (20.9)
10–19.9	205 (34.3)	96 (30.1)	109 (39.2)
20–29.9	72 (12.1)	24 (7.5)	48 (17.3)
30–39.9	17 (2.8)	0 (0.0)	17 (6.1)
>40	4 (0.7)	0 (0.0)	4 (1.4)
Unknown	42 (7.0)	0 (0.0)	42 (15.1)
**Mode of transportation**	**Total**	**Platform**	**Non-Platform**
Taxi	478 (80.2)	266 (83.4)	212 (76.5)
Public bus	25 (4.2)	14 (4.4)	11 (4.0)
Private car	53 (8.9)	21 (6.6)	32 (11.6)
Ambulance	40 (6.7)	18 (5.6)	22 (7.9)
Not available	1	0	1
**Accompanying person**	**Total**	**Platform**	**Non-Platform**
Husband	296 (49.8)	162 (51.1)	134 (48.4)
Another relative	227 (38.2)	116 (36.9)	109 (39.7)
No one	71 (12.0)	38 (12.0)	33 (11.9)
Not available	3	2	1
**Accompanying midwife**	**75 (12.6)**	**45 (14.1)**	**30 (10.8)**

Data presented as number (percent). Platform facilities were significantly closer in proximity to GARH than were non-Platform facilities (p<0.01).

#### Labor characteristics and referral indications

There were 261 (43.7%) antenatal, 336 (55.9%) intrapartum, and 2 (0.3%) postpartum referrals, the distribution of which was similar across Platform and non-Platform sites. The indications for referral are shown in Table 3. The majority of referred patients arrived with a referral slip (404; 67.7%); this did not differ between Platform (227; 71.2%) and non-Platform (177; 63.7%) facilities. In the majority, a pre-referral phone call was not made [overall 204 (37.2%); Platform 115 (39.2%); non-Platform 89 (34.8%)]. Pre-referral treatment was significantly more common among Platform facilities, albeit low [overall 129 (25.1%); Platform 81 (28.5%); non-Platform 48 (21.0%) (p = 0.05)]. Among the referred patients, 588 (99.8%) delivered at GARH; 371 (63.4%) had vaginal and 214 (36.6%) had cesarean deliveries, consistent among Platform and non-Platform referrals.

**Table 3:** Indications for referral.

Indication	Total	Platform	Non-Platform
Labor dystocia^a^	192 (26.2)	115 (29.7)	77 (22.3)
Hypertensive disorders^b^	90 (12.3)	54 (14.0)	36 (10.4)
Prior uterine scar^c^	85 (11.6)	48 (12.4)	37 (10.7)
Maternal miscellaneous^d^	60 (8.2)	33 (8.5)	27 (7.8)
Self-referral	49 (6.7)	15 (3.9)	34 (9.8)
Anemia^e^	42 (5.7)	23 (5.9)	19 (5.5)
Fetal compromise^f^	42 (5.7)	23 (5.9)	19 (5.5)
Prematurity^g^	35 (4.8)	13 (3.4)	22 (6.4)
Fetal malpresentation^h^	27 (3.7)	12 (3.1)	15 (4.3)
Rupture of membranes^i^	25 (3.4)	14 (3.6)	11 (3.2)
Labor	23 (3.1)	8 (2.1)	15 (4.3)
Acute hemorrhage^j^	13 (1.8)	6 (1.6)	7 (2.0)
Multiple gestation^k^	11 (1.5)	7 (1.8)	4 (1.2)
Ectopic or miscarriage	8 (1.1)	2 (0.5)	6 (1.7)
Previous poor obstetric outcome^l^	8 (1.1)	4 (1.0)	4 (1.2)
Lack of resources at referral site^m^	6 (0.8)	1 (0.3)	5 (1.4)
Infectious causes^n^	5 (0.7)	3 (0.8)	2 (0.6)
Maternal age extremes (> 35 years)	5 (0.7)	2 (0.5)	3 (0.9)
Intra-uterine fetal demise	4 (0.5)	2 (0.5)	2 (0.6)
Fetal miscellaneous^o^	2 (0.3)	1 (0.3)	1 (0.3)
No/poor prenatal care	1 (0.1)	1 (0.3)	0 (0.0)
**Total**	**733**	**387**	**346**
One referral indication	467 (78.0)	255 (80.0)	212 (76.0)
Two referral indications	124 (21.0)	60 (19.0)	64 (23.0)
Three referral indications	6 (1.0)	4 (1.0)	2 (1.0)
Agreement in referral indication and admitting diagnosis	440 (73.7)	240 (75.2)	200 (71.9)

There were 597 referral records captured for deliveries occurring at the Greater Accra Regional Hospital from September 1, 2017 to November 30, 2017. There were no differences in the number of referral indications or in agreement in referral indication and admitting diagnosis between Platform and non-Platform facilities.

a. Cephalopelvic disproportion, fetal macrosomia, large maternal abdomen, post-term pregnancy, over 40 weeks estimated gestational age, borderline pelvis, contracted pelvis, delayed or prolonged labor, arrest of labor, slow progress, failed induction, unfavorable cervix, high head in labor, obstructed labor.

b. Chronic hypertension, PIH, pre-eclampsia, severe pre-eclampsia, or eclampsia.

c. Previous cesarean delivery, prior myomectomy, or previous uterine rupture.

d. Maternal asthma, diabetes, gestational diabetes, prior abdominal surgery, uterine fibroids, vaginal/vulvar growth or discharge, proteinuria, urinary tract infection, fever, generalized edema, short/long pregnancy interval, short maternal stature, maternal distress, sterilization request, grand multiparty, seizure disorder, mental illness, obesity, patient refusal for care, patient lacks laboratory or scan information, crippled, rhesus negative.

e. Maternal anemia or sickle cell disease.

f. Abnormal cardiotocography, fetal tachycardia, fetal distress, oligohydramnios, meconium stained amniotic fluid, decreased fetal movement, intrauterine growth restriction, umbilical cord prolapse, chorioamnionitis, maternal fever.

g. Prematurity (gestation < 37 weeks), preterm labor or preterm premature rupture of membranes.

h. Face/mentoposterior, brow, breech/footling breech, oblique, transverse, unstable lie, arm prolapse, leading twin breech, compound presentation.

i. Rupture of membranes, loosing liquor, prolonged rupture of membranes, premature rupture of membranes (rupture without labor with gestation ≥ 37 weeks)

j. Placenta previa, placental abruption, placenta accreta, ante-, intra- and postpartum bleeding, uterine rupture, unclassified hemorrhage.

k. Twin pregnancy, triplet pregnancy.

l. Bad obstetric history, prior stillbirth, prior ectopic pregnancy, unexplained history of intrauterine fetal death, previous failure to progress, prior cervical cerclage, previous peripartum hemorrhage.

m. No electricity, no bed, no gloves, no water, no doctor, no anesthetist.

n. Hepatitis B, malaria, syphilis, human immunodeficiency virus.

o. Anencephaly, severe hydrocephalus, polyhydramnios, fetal deformity.

#### Urgent referrals

Five hundred fifteen (86.3%) patients were deemed urgent referrals for emergency conditions; the most frequent were labor dystocia (189; 29.8%), hypertensive disorders of pregnancy (90; 14.2%), prior uterine scar (59, 9.3%), maternal comorbidities (46, 7.3%), and fetal compromise (42, 6.5%) without variation by facility type. Urgent indications accounted for all but one of the midwife accompanied referrals shown in Table 2. The mode of delivery for urgent cases did not differ from that stated overall.

The median (IQR) referral to arrival time for urgent referrals is shown in Table 4. When data were disaggregated by mode of transportation, arrival time was significantly shorter for those arriving by ambulance (p = 0.018). Referrals that were accompanied by a midwife also resulted in significantly shorter median referral to arrival times in Platform (60 min) vs non-Platform (135 min) hospitals (p = 0.003). Only 140 (23.5%) patients referred urgently arrived within 2 hours with 85 (30.1%) from Platform and 55 (22.7%) from non-Platform sites (p = 0.059). Of the 74 urgent referrals accompanied by a midwife or nurse, 44 (59.5%) arrived within 2 hours.

**Table 4:** Referral to arrival time for urgent referrals.

**Referral to Arrival Time**	**Total N = 515**	**Platform N = 282**	**Non-Platform N = 233**
Median (IQR) min	293 (111–1887)	293 (91–2160)	296 (129–1675)
**By Time of Day**			
Day (08:00–19:59)	283 (126–2160)	288 (107–1955)	271 (134–2607)
Night (20:00–07:59)	356 (90–1795)	321 (78–2377)	385 (106–1074)
**By Mode of Transportation**	**Total**	**Platform**	**Non-Platform**
Taxi	337 (124–2334)	323 (110–2459)	390 (130–2051)
Public van	1531 (202–10410)	971 (80–4023)	3925 (689–10860)
Private Car	237 (80–1025)	219 (80–1464)	244 (120–721)
Ambulance	135 (65–300)*	69 (41–347)	240 (102–300)
**By Midwife Accompanied**	**Total**	**Platform**	**Non-Platform**
Accompanied by midwife/nurse	80 (41–272)	60 (30–168)†	135 (75–356)

Data presented as median (IQR). Ambulance was a significantly faster than other modes of transport (*p = .018), but there was no difference between Platform and non-Platform hospitals. Referral to arrival time significantly decreased when Platform patients were accompanied by a midwife (†p = .0034).

#### Outcomes observed in referral analysis

Maternal and fetal outcomes are shown in Table 5. There were 30 stillbirths; four were known prior to transfer, 10 occurred during the referral process, 14 had a fetal heartbeat on arrival at GARH and in two the fetal heart rate was not recorded. In these, the referral to arrival times varied widely, from 20 min to several days. One woman was referred for failure to progress and fetal distress; however, on arrival she was diagnosed with uterine rupture and intrauterine fetal demise. For this tragic case, the referral to arrival time was 28 minutes by taxi and the woman was accompanied by a midwife. Fortunately, the woman survived. There was 1 (0.2%) maternal death, which occurred within 24 hours of hospital arrival. She was referred with hypertensive disorder of pregnancy from a non-Platform hospital, but received treatment prior to transfer. She arrived in 132 minutes by ambulance accompanied by a midwife; the fetus died during transport.

**Table 5:** Outcomes.

**Maternal Outcome**	**Total**	**Platform**	**Non-Platform**
Discharged without complications	495 (84.5)	264 (83.8)	231 (85.2)
Discharged after prolonged hospitalization	90 (15.4)	51 (16.2)	39 (14.4)
Death	1 (0.2)	0 (0.0)	1 (0.4)
Not available	11	4	7
**Fetal Heart Rate on Arrival**	Total	**Platform**	**Non-Platform**
Present	572 (97.6)	305 (96.8)	267 (98.5)
Absent	14 (2.4)	10 (3.2)	4 (1.5)
Not available	11	4	7
**Fetal Outcome**	**Total**	**Platform**	**Non-Platform**
Live Birth	555 (93.8)	298 (94.0)	257 (93.5)
Stillbirth	30 (5.1)	18 (5.7)	12 (4.4)
Ectopic	7 (1.2)	1 (0.3)	6 (2.2)
Not available	5	2	3
APGAR 1 min	5.9 ± 2.1	5.8 ± 2.2	6.1 ± 2.1
APGAR 5 min	7.2 ± 2.3	7.1 ± 2.3	7.4 ± 2.4
Baby Weight	3.0 ± 0.9	3.0 ± 0.8	2.9 ± 1.0
Range	0.3–5.0	0.3–5.0	0.5–5.0

Data presented as mean ± SD or number (percent). There are no differences in outcomes.

## Discussion

Weak referral systems result in significant delay in the management of obstetric emergencies in many countries [9, 27, 28]. Studies frequently describe factors that lead to referral delay or report problems associated with delay, but few quantitate delay or explore locally driven solutions to address it [27, 28]. This study contributes to the literature in both regards.

This feasibility project utilized WhatsApp to improve communication regarding emergency obstetric referrals in Accra, Ghana and was endorsed by the Greater Accra Regional Director of the Ghana Health Service. Indeed, uptake of the Kybele Referral Platform was rapid; 618 cases were posted within three months and the participant number increased from 69 to 81 during that time. Use has been sustained; at the time of this writing there are 174 individual Platform users. Our working theory of change has been supported. We hypothesized that this low-cost intervention would be readily accepted by providers, and that it could be done in a way that protected patient privacy. The anonymity of the WhatsApp platform made it impossible for us to trace women through the referral process, however, the fact that the platform remains actively used nearly five years later is strong affirmation that the intervention was not only feasible, but helpful to the community of providers in Accra.

In our subsequent analysis of referrals to GARH, it is encouraging that pre-referral treatment was seen more commonly in Platform facilities and referral to arrival times were shortest when urgent cases were transferred by ambulance and when accompanied by a midwife, although attribution of these findings to the Platform cannot be made. The analysis of the Platform transcripts revealed that pre-referral treatment was initiated in 55.2% of posted cases, yet in the referral dataset, pre-referral treatment was provided for only 28.5% of Platform facility patients. It was not mandatory that every patient being referred be posted on the Platform, but when cases were posted and viewed across a spectrum of users, pre-referral treatment may have improved due to higher visibility and scrutiny of the care provided. Alternatively, sicker patients may have been more likely to be posted and therefore more likely to need pre-referral treatment. As has been similarly reported, we found that irrespective of facility category, most high-risk obstetric referrals were unaccompanied by healthcare staff, contrary to national guidelines [2, 6, 29, 30]. When accompanied, we found that the referral to arrival time significantly decreased and this was most pronounced in Platform facilities.

The Platform highlighted failure in the promotion of care along the recommended referral pathway. Platform analysis showed that only 19% of posted cases were received by district hospitals, whereas GARH received 61%. The referral data similarly found that 14% of referrals to GARH came from district hospitals, while 62% came directly from lower-level facilities. Ideally, district-level hospitals should be acting as the intermediary in receiving lower acuity cases, to reduce the high volume of referrals to tertiary hospitals. Bailey and colleagues studied obstetric referral patterns and facility readiness among 977 delivery centers across Ghana [3]. They found that district hospitals accounted for 52% of deliveries and 9% of referrals [3]. Limitations of the Bailey study included no knowledge of where women went once referred or the timeliness of referral, both of which this study addresses. These are important considerations for the ongoing efforts to decongest high-volume referral hospitals.

This work further demonstrates that in the urban setting of Accra, Ghana, significant delay occurs in the obstetric referral process, even among hospitals that were included on the Platform. Research on emergency obstetric referrals in Ghana and other countries is limited, particularly in the quantitative measure of delay in reaching secondary and tertiary levels of care. A study in Mozambique reported that transport and referral delay accounted for 60% of the 712 maternal deaths but details on timeliness were not provided [13]. In Rwanda, neonatal outcomes were significantly worse among mothers whose travel times exceeded 90 min [10]. In the Upper East Region of Ghana, 104 maternity unit officers responded to an oral questionnaire on emergency obstetric interventions and reported a 37 min (range 5–120 min) mean travel time to the nearest referral facility using various means of transport; however, travel time was not measured [4]. Obtaining quantitative data from interviews is subject to recall bias and could be inaccurate. Twenty years ago, Nkyekyer reported on peripartum referrals to KBTH, but timeliness was inadequately assessed [29]. Data were available for 46 (11.6%) of 396 referred patients, and the average transport time to reach KBTH was 78 min. The time from referral decision until facility departure was determined for seven women but was not stated [29]. Our study measured the time interval between referral and arrival at the receiving hospital and provides important detail to a recognized gap in the literature. We found that among the 515 urgent referrals, the median referral decision to arrival time was just under five hours, clearly exceeding the two-hour recommended time interval.

Delay is influenced by more than just the physical distance to a health facility and women in Accra typically do not report “distance” to a health facility as a barrier to care [1]. Many patients represented in the current study arrived from facilities within 20 km of GARH yet still had long referral to arrival times. It is uncertain what happens to women in this time interval. A recent study from Lagos, Nigeria might shed some light [31]. Forty-seven postpartum women residing in the metropolitan area were interviewed regarding timeliness in reaching a hospital from home. Travel times of 5–240 min varied by traffic congestion during the day and security issues and lack of public transport at night [31]. Women reported making stops along the way to pick up support persons and to purchase items deemed necessary for hospitalization. Seventeen of the 47 women were referrals to a CEmOC facility when the initial facility could not provide the necessary care. When women were referred, they were often expected to determine on their own how to reach the referral facility, which prolonged delay and increased cost. The authors report that, despite recognizing danger signs and referral, “pregnant women are faced with conundrums on ‘when’, ‘where’ and ‘how’ to reach CEmOC facilities” [31].

These conditions were likely present in the current study, as we found that 80.2% of referred patients arrived by means of public transport, which did not improve among Platform facilities, and as corroborated by others [2, 3, 6, 18, 30, 32]. This is concerning given that in 2004 Ghana launched a national ambulance service that has been expanded, especially in urban areas [3]. When ambulances are utilized, delay can still occur as patients may need to wait for ambulances located remotely or provide funds to fuel the vehicle [2, 4–6, 29, 30]. However, when traveling by ambulance, the median referral to arrival time was significantly shorter. Nevertheless, the public may perceive that taxis are a faster form of transport than ambulances and this needs to be addressed [33]. In 2002, Nkyekyer found that 65.8% of referred patients utilized taxis or small public buses for transport [29]. In 20 years, little has changed.

In the present study, the leading referral indications were labor dystocia (26.2%), hypertensive disorders of pregnancy (12.3%), and prior uterine scar (11.6%). This is in agreement with other reports, which have similarly found obstructed labor to be the most common referral indication [3, 13, 22, 23, 31]. In Nkyekyer’s 2002 report, only 2% of patients were referred for prior uterine scar [29]; however, this indication has substantially increased [32]. A rising cesarean delivery rate, particularly in Accra, may worsen the existing burden on CEmOC hospitals [1, 30]. A better understanding of labor management practices in lower-level facilities is needed to stem the tide of increasing surgical deliveries [13].

There are several limitations of the current study. We had hoped to gather data on all referred obstetric patients during the September to November 2017 data collection period; however, we captured data for only 597 (47.5%). This was likely due to chart unavailability, data collector unavailability or inattentiveness, and other undetermined factors. Second, we had hoped to gather data on referrals prior to initiating the Platform to allow for a baseline comparison of Platform vs non-Platform facilities, but data collected prior to March 2017 were incomplete and poor quality. Therefore, we could only analyze data collected after the Platform was initiated limiting the attribution of findings. Third, we could not identify individual patients on the Platform for privacy reasons and were therefore unable to determine the arrival times for posted cases. For this reason, specific case outcomes were also unavailable. Fourth, we could have surveyed the referred women to describe their experience, especially in relation to reasons for the delays. For example, we could have queried women as to why they did not report to GARH at time of referral. Indeed, we found some referral to arrival time intervals that spanned several weeks, which is why we focused on urgent referrals. Fifth, delay almost certainly impacted maternal and newborn outcomes, but we were unable to ascertain whether this delay was most significant before leaving the initial site, in route, or both. It would have been useful to gather more information from the source facilities, such as time of departure and reasons responsible for referral delay. Other studies, however, have described these factors which include needing to collect money for treatment or transport, waiting for relatives, needing permission from family members, patient preferences for traditionalist and spiritual practices, first refusal of referral due to fear of disrespect, medical procedures, or surgery, waiting for transport, and traffic congestion [1–3, 5, 6, 13, 27, 28, 30, 34–36]. In some cases, pregnant women are referred from one facility to another on the basis of no bed availability or lack of resources [30, 31, 37]. Fortunately, in the present report, this occurred for only 6 (0.8%) patients.

One advantage to our approach in determining delay from the time of referral decision is that delay likely extends beyond the time required for transport alone. The Platform was helpful in that it provided the receiving hospital advanced warning in order to make preparations to receive a compromised patient. Lack of advanced warning that a patient is coming and omitting a referral slip have been shown to negatively impact preparedness and outcomes at the receiving hospital [2, 3, 6, 34]. Caution in the use of applications such as WhatsApp for healthcare must be exercised. It is paramount to establish guidelines to ensure patient privacy, data security, and phone stewardship [21]. In the current study, participants were forbidden to share photographs or patient identifiers and were reprimanded for posting unrelated content.

It is important to identify and quantify referral-related barriers in the provision of timely CEmOC in low resource countries and this topic remains vastly under-researched [2, 6, 34, 35]. A strong referral system needs communication and coordination among all stakeholders along the referral pathway with tailored referral protocols, cost absorption and efficient transportation to reduce stillbirth, neonatal and maternal deaths [2, 6, 11, 18, 36].

## Conclusion

The inability of high-risk women to access CEmOC remains a major challenge in addressing the global burden of maternal and newborn mortality. This study supports the feasibility of using WhatsApp mobile messaging technology to address communication and coordination along the referral pathway while highlighting that referral delays remain problematic. Our analysis indicates that further study and planning is warranted to optimize obstetric referrals in Accra, Ghana. As countries seek to accelerate mortality reduction to meet the Sustainable Development Goal targets, referral systems need further consideration in order to ensure appropriate access, quality and timeliness of care.
